# Impact of a hospital-wide hand hygiene promotion strategy on healthcare-associated infections

**DOI:** 10.1186/2047-2994-1-13

**Published:** 2012-03-23

**Authors:** Moi Lin Ling, Kue Bien How

**Affiliations:** 1 Infection Control, Singapore General Hospital, Outram Road, 169608 Singapore

**Keywords:** hand hygiene, healthcare-associated infections, system change

## Abstract

**Background:**

During the Severe Acute Respiratory Syndrome (SARS) outbreak, high compliance in healthcare workers to hand hygiene was primarily driven by fear. However, the post-SARS period confirmed that this practice was not sustainable. At the Singapore General Hospital, a 1,600-bedded acute tertiary care hospital, the hand hygiene program was revised in early 2007 following Singapore's signing of the pledge to the World Health Organization (WHO) "Clean Care is Safer Care" program.

**Findings:**

A multi-prong approach was used in designing the hand hygiene program. This included system change; training and education; evaluation and feedback; reminders in the workplace; and institutional safety climate. Hand hygiene compliance rate improved from 20% (in January 2007) to 61% (2010). Improvement was also seen annually in the compliance to each of the 5 moments as well as in all staff categories. Healthcare-associated MRSA infections were reduced from 0.6 (2007) to 0.3 (2010) per 1000 patient-days.

**Conclusions:**

Leadership's support of the program evidenced through visible leadership presence, messaging and release of resources is the key factor in helping to make the program a true success. The hospital was recognised as a Global Hand Hygiene Expert Centre in January 2011. The WHO multi-prong interventions work in improving compliance and reducing healthcare associated infections.

## Findings

The WHO Multimodal Hand Hygiene Improvement Strategy comprising a Guide to Implementation and a range of tools constructed to facilitate implementation of each component was used in a 1600-bedded acute tertiary care general hospital in Singapore [1]. The objective is to change healthcare workers' behavior and improve hand hygiene compliance. During the Severe Acute Respiratory Syndrome (SARS) outbreak in 2003, a high compliance of close to 90% in healthcare workers to hand hygiene was achieved. However, this was primarily driven by fear for transmission of pathogens to self. Following the closure of the SARS outbreak globally, we noted that the hand hygiene compliance decreased to that of the baseline before the SARS outbreak i.e. the high hand hygiene compliance was not sustainable.

The hand hygiene program was revised in early 2007 following Singapore's signing of the pledge to the World Health Organization (WHO) "Clean Care is Safer Care" program.

The multi-prong approach used includes:

1. System change:

a. Alcohol based hand rub was promoted for routine use of hand hygiene instead of the previous 4% chlorhexidine handwash. During SARS, alcohol hand rub bottles were installed at the foot of every patient bed and all lift lobbies in an attempt to enable easy access by healthcare workers to hand hygiene products. From 2007, more alcohol hand rub bottles were installed along ward corridors and near food establishments in the hospital ground.

b. Following the review of the WHO Hand Hygiene Manual (Advanced Draft 2006) [2], the Infection Control Committee approved the use of at least either ethanol 80% (v/v) or isopropyl alcohol 75% (v/v) in the procurement of hand hygiene alcohol hand products. This implies the use of a higher content of alcohol than before. From July 2009, 4% chlorhexidine handwash agent bottles were removed from the clinical areas except for the Operating Theatres, Endoscopy Unit and treatment rooms. This was done to reduce the incidence of dryness or skin irritation resulting from concomitant use of both alcohol and chlorhexidine [1]. Hand moisturizer was provided freely for healthcare workers' use. They are encouraged to use it as often as possible to protect their hands.

**Figure 1:**
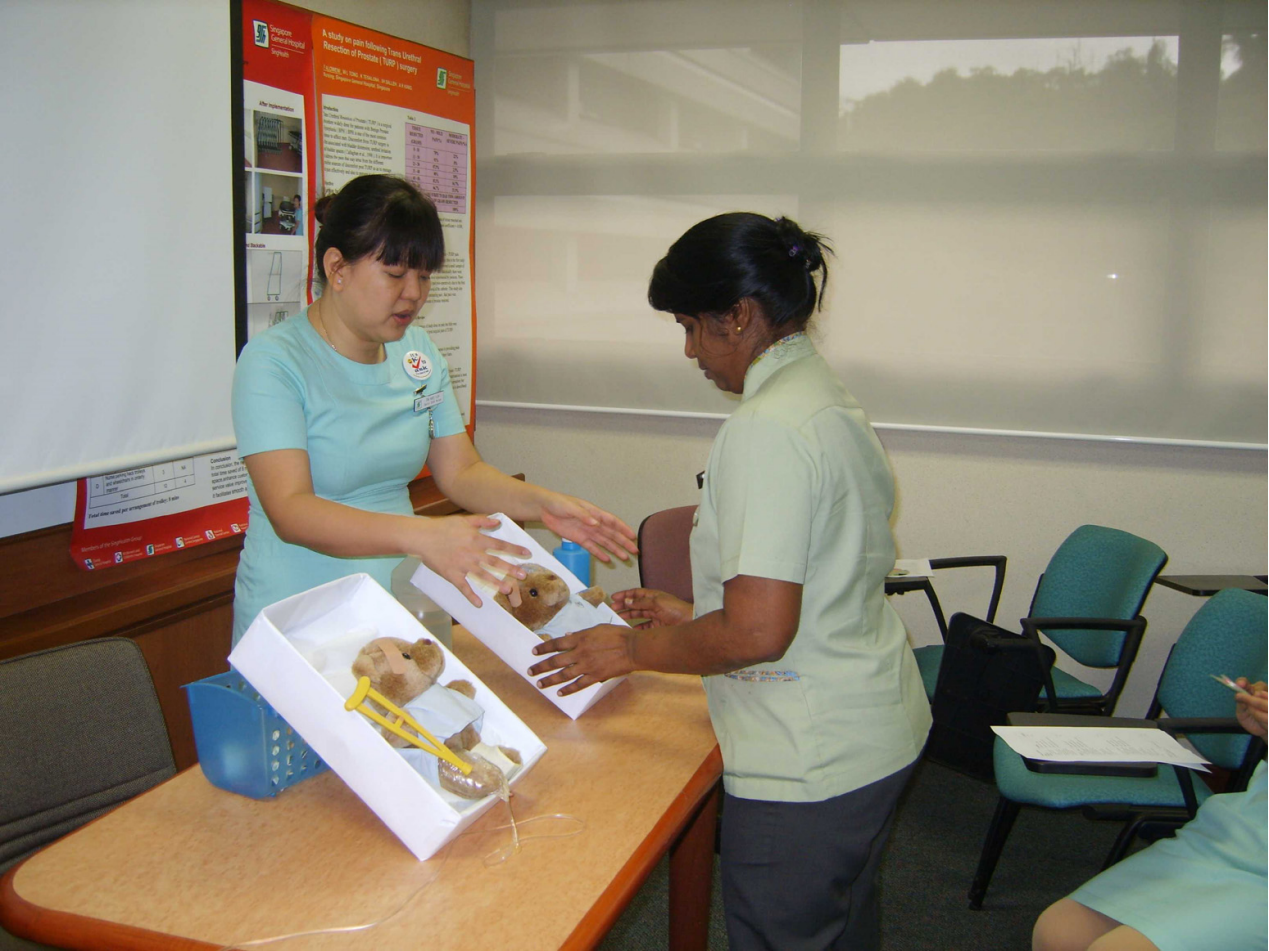
**Teddy bears dusted with Glo-germ were used as patient models in teaching healthcare workers the importance of hand hygiene as well as the WHO 5 moments**.

2. Training & education:

a. WHO training DVDs were used to illustrate clinical scenarios of hand hygiene opportunities to all healthcare workers. Although these were in French, they provided clear teaching on the 5 moments to the staffs.

b. Creative educational tools were also used to teach healthcare attendants and junior nurses. Teddy bears dusted with Glo-germ were used as patient models in teaching healthcare workers the importance of hand hygiene as well as the WHO 5 moments (Figure 1).

c. Powerpoint slides with detailed explanation of the 5 moments were created for doctors, nurses and allied health. These were uploaded on the hospital intranet for easy access by the staffs.

**Figure 2:**
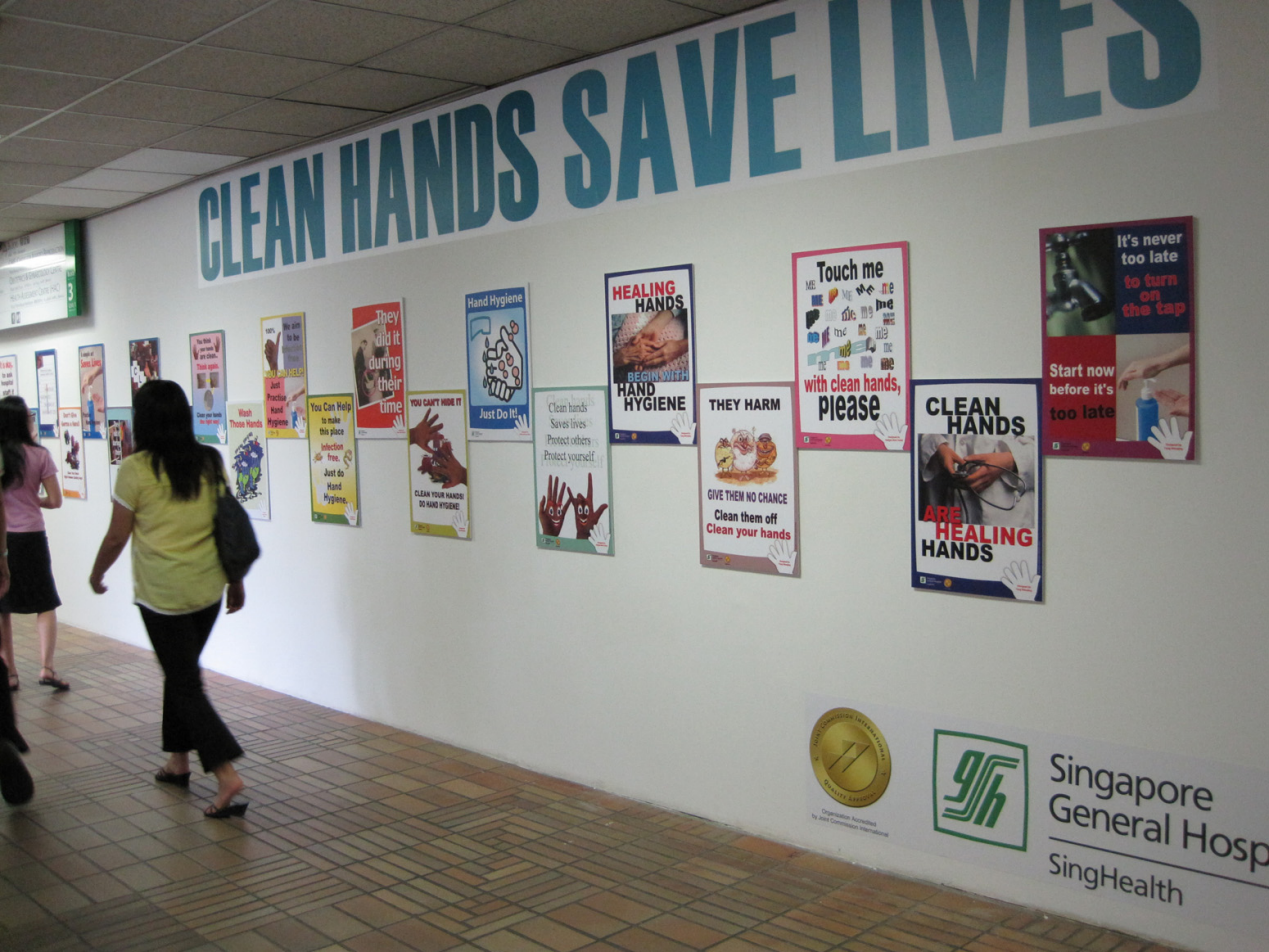
**Posters at walkways**.

**Figure 3:**
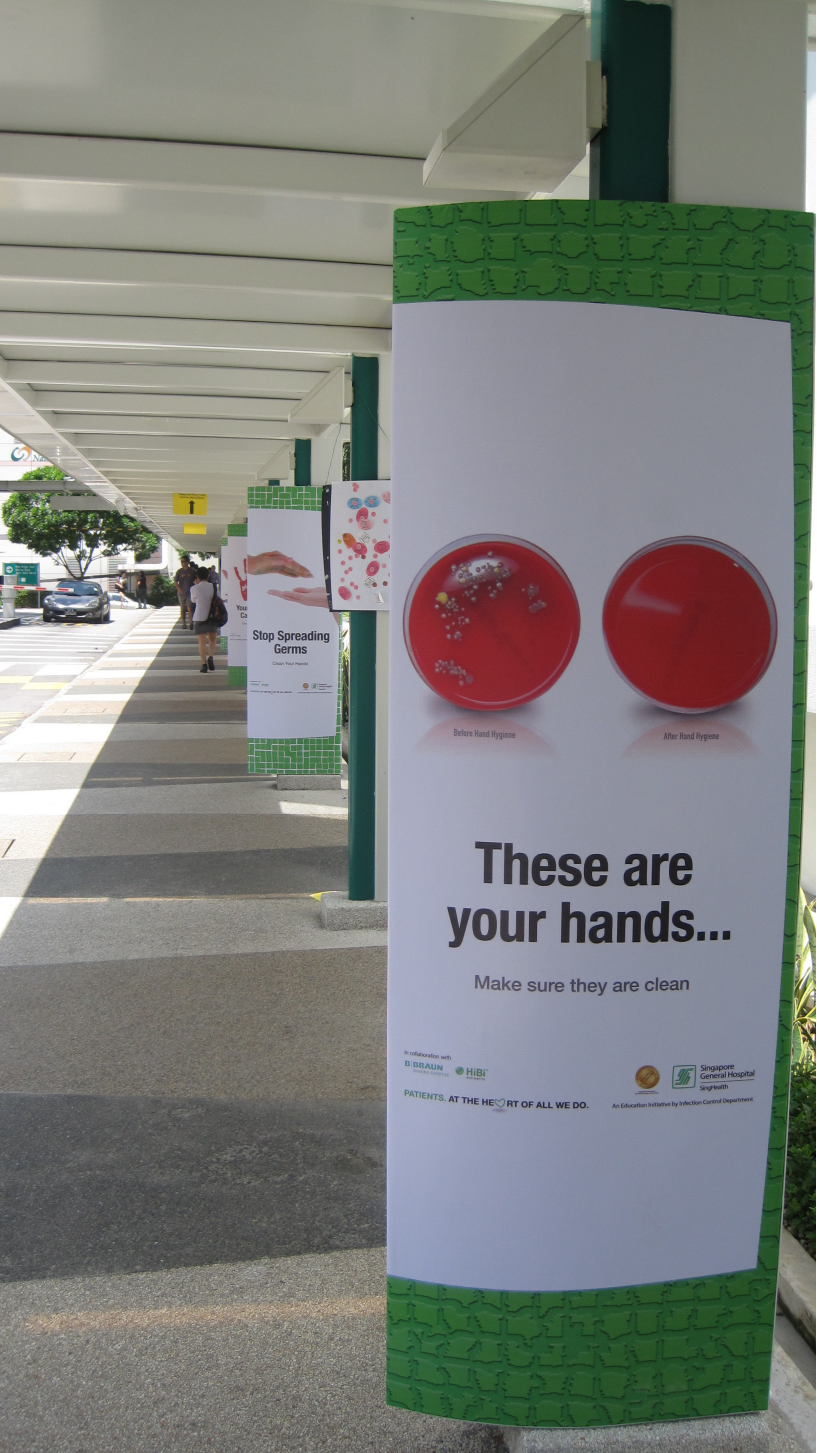
**Posters at linkway to train station**.

**Figure 4:**
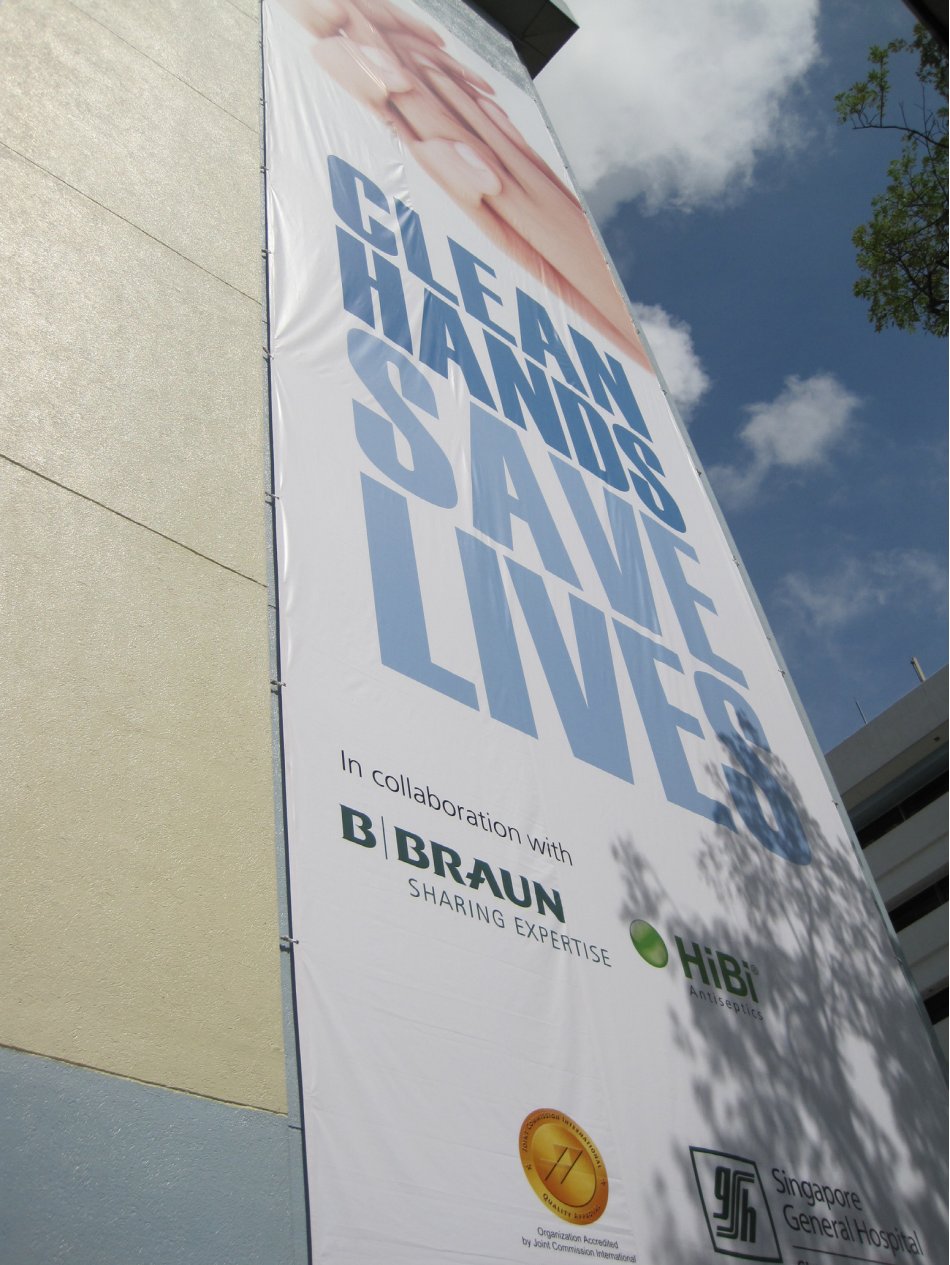
**Giant poster display 2009 spanning nine floors**.

**Figure 5:**
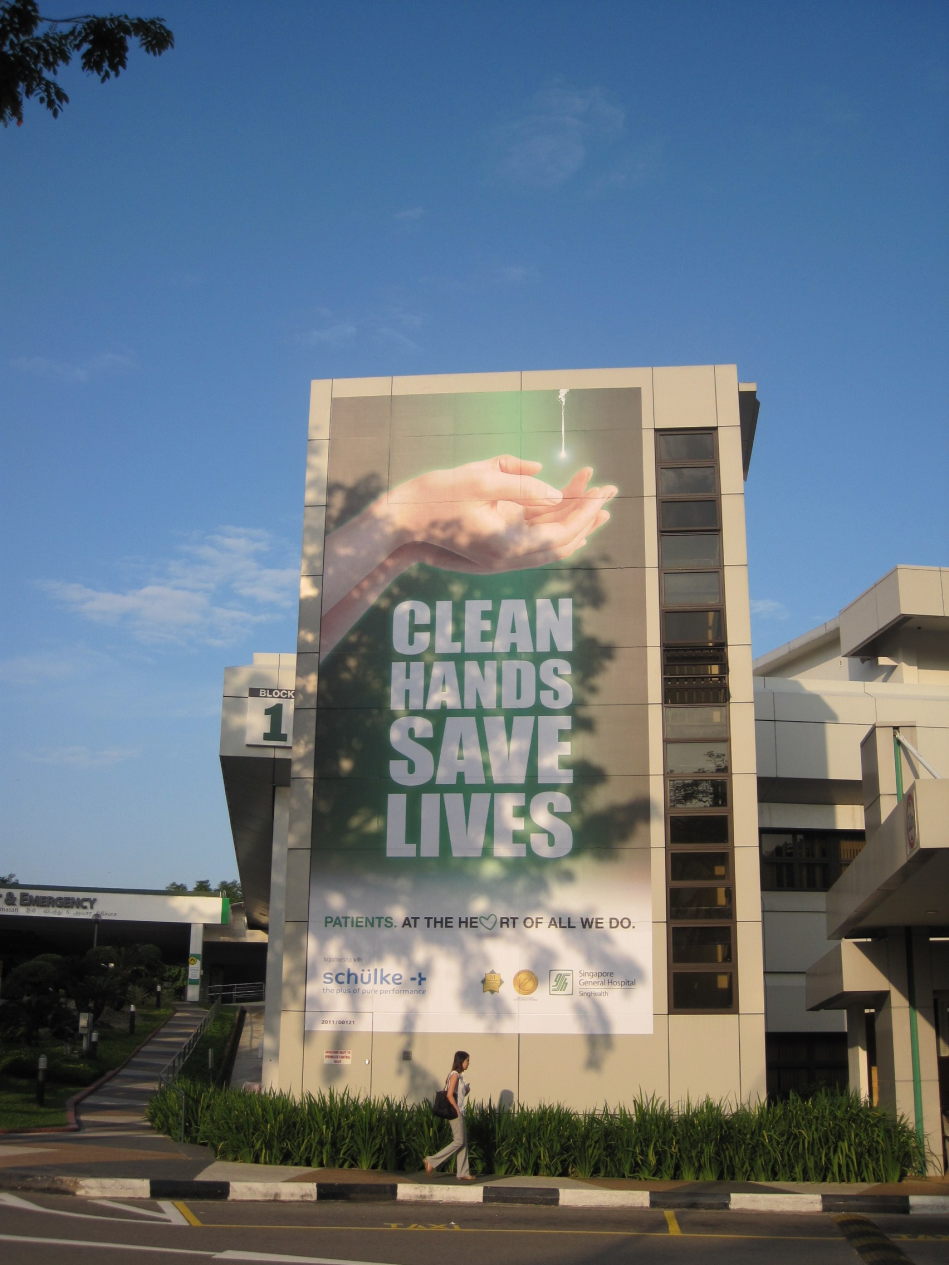
**Giant poster display 2010**.

**Figure 6:**
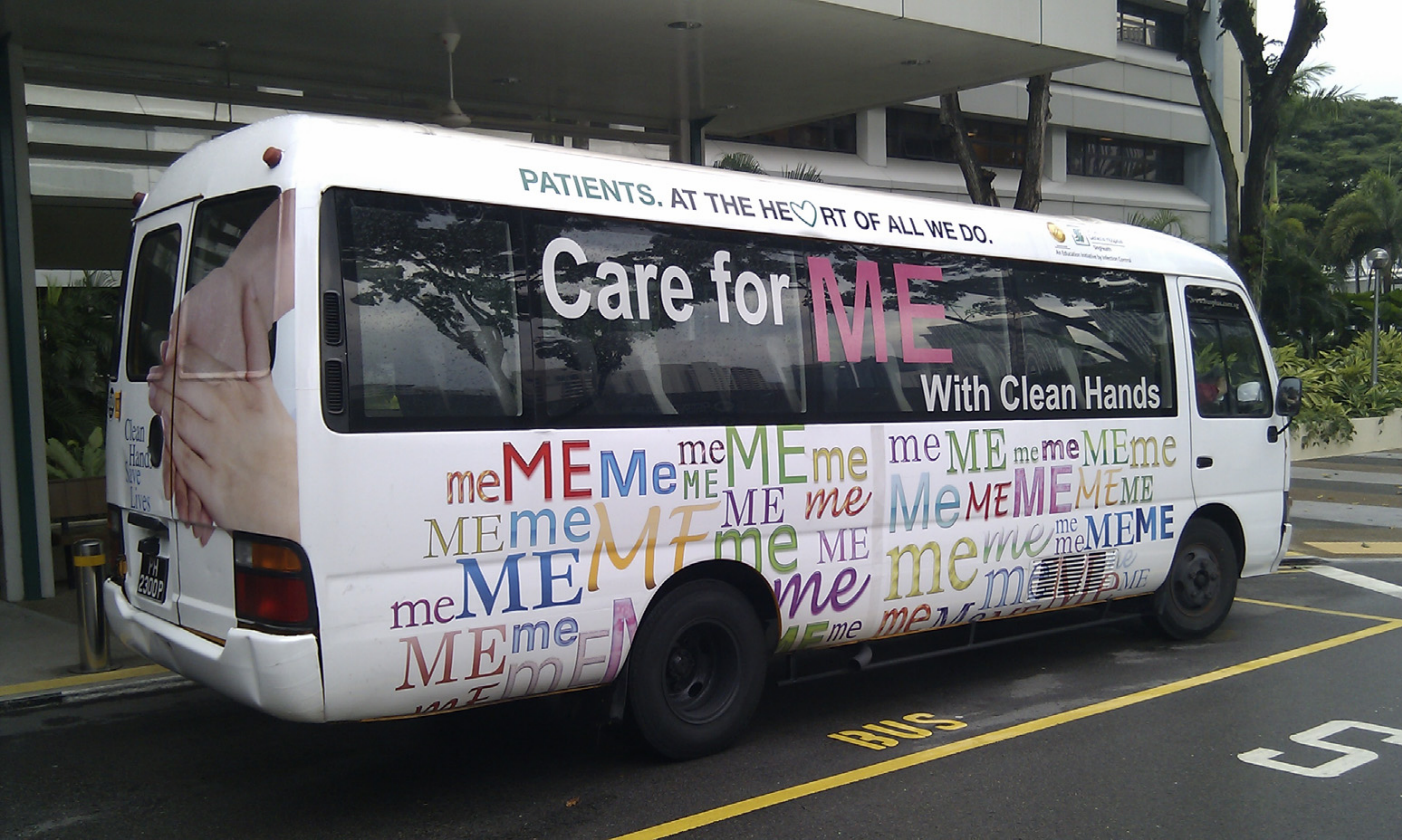
**Hand hygiene messages on shuttle buses plying the hospital campus**.

**Figure 7:**
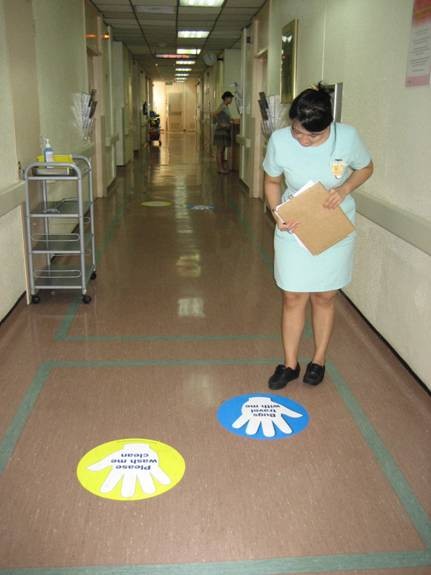
**Hand hygiene messages on floor stickers in wards**.

**Figure 8:**
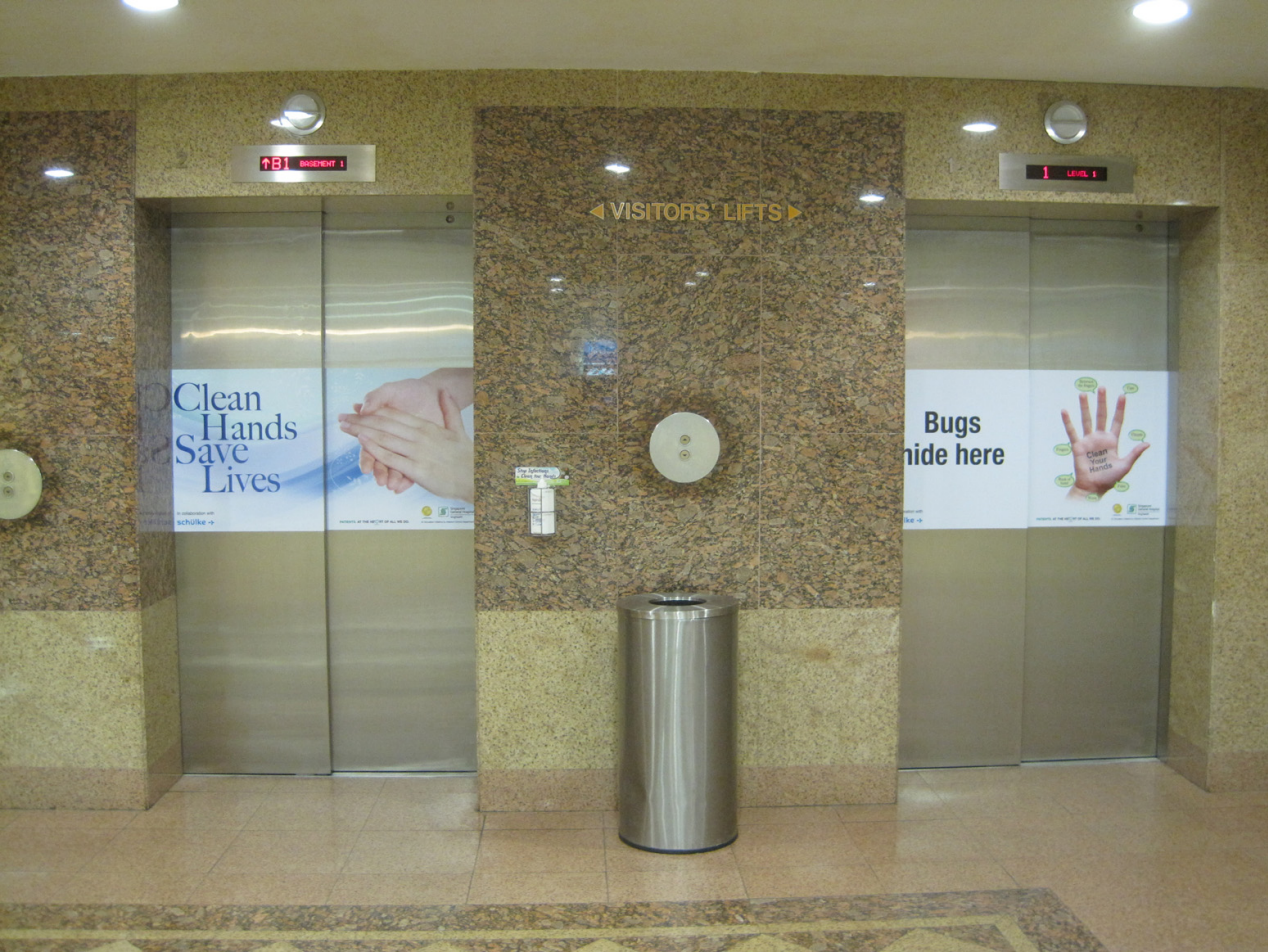
**Hand hygiene messages on doors of public lifts**.

**Figure 9:**
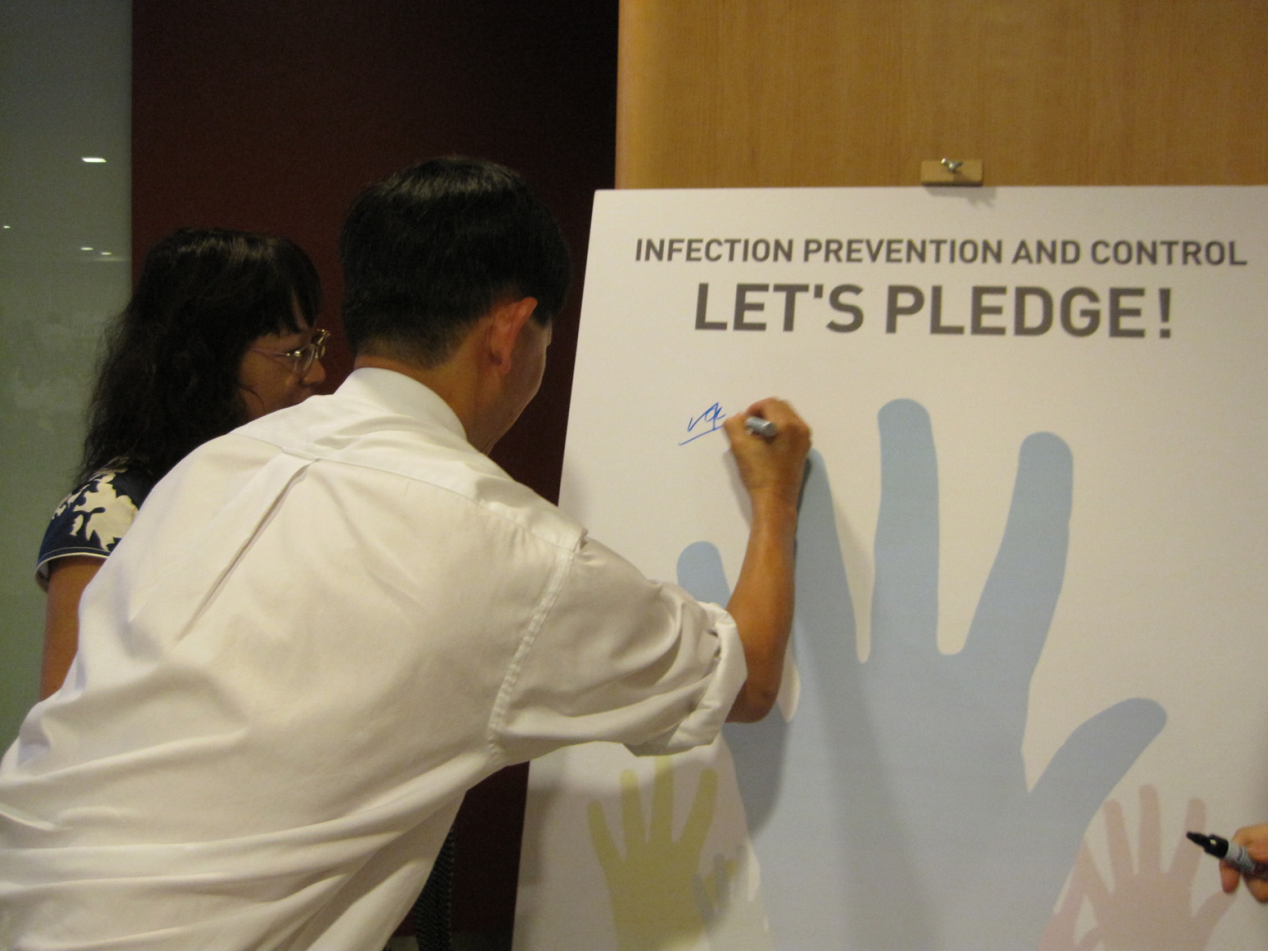
**Hospital CEO signing pledge of commitment to hand hygiene program**.

3. Evaluation and feedback: Feedback surveys are conducted annually amongst staffs to gather feedback and comments on products used or issues faced during practice. The last survey done in November 2010 confirmed that the use of posters have helped to remind staffs and public on the messages of hand hygiene.

4. Reminders in the workplace: More than 150 posters were designed from March 2007 for display at lifts and walkways (Figures 2 and 3). Giant posters on hand hygiene messaging were designed to convey the hospital's commitment to the public (Figures 4 and 5). Shuttle buses, floor surfaces and lift doors are used to display reminders to both staffs and public (Figures 6, 7 and 8).

5. Institutional safety climate: Leadership's support and commitment was clearly visible at events and meetings. The Chief Executive Officer (CEO) led the hospitals staffs in November 2009 in a pledge of commitment to the Hand Hygiene Program (Figure 9).

**Figure 10:**
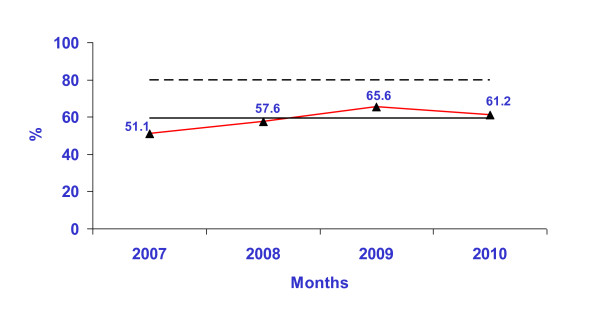
**Annual hospital hand hygiene compliance rate from 2007 - 2010**.

**Figure 11:**
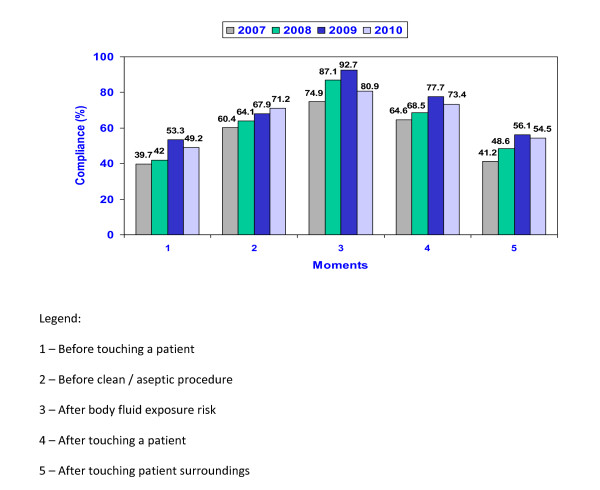
**Hand hygiene compliance rate by WHO 5 Moments from 2007-2010**.

**Figure 12:**
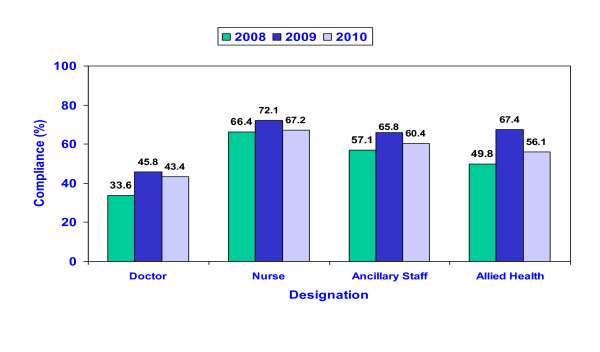
**Hand hygiene compliance rate by designation from 2008-2010**.

Hand hygiene compliance rate improved from 20% (in January 2007) to 61% (2010) (Figure 10). Improvement was also seen annually in the compliance to each of the 5 moments as well as in all staff categories (Figure 11 and Figure 12). Although hand hygiene compliance was lowest in the doctors category, it is encouraging to note that improvement was also seen year to year in this job category (Figure 12).

The hand hygiene program was an integral part also of an ongoing MRSA reduction program in the hospital, which includes the implementation of the MRSA bundle. The "MRSA bundle" includes five components of care. This bundle was first introduced by the Institute of Healthcare Improvement (IHI) in 2006 in their "Protecting 5 Million Lives from Harm" campaign. It comprised the following elements [3]:

1. Hand Hygiene

2. Decontamination of the environment/equipment

3. Active surveillance cultures

4. Contact precautions for infected and colonized patients

5. Device bundles (central line and ventilator bundles)

**Figure 13:**
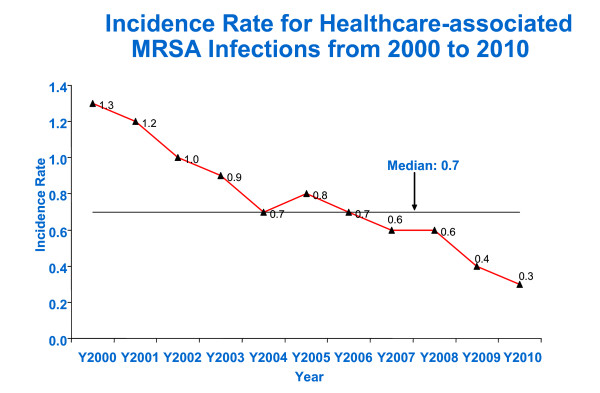
**Incidence rate for healthcare-associated MRSA infections**.

Most of the elements were routine practices in the hospital except for active surveillance cultures, which were implemented hospital-wide for high risk patient groups from 2008. Healthcare-associated MRSA infections were noted to reduce from 0.6 (2007) to 0.3 (2010) per 1000 patient-days (Figure 13).

**Figure 14:**
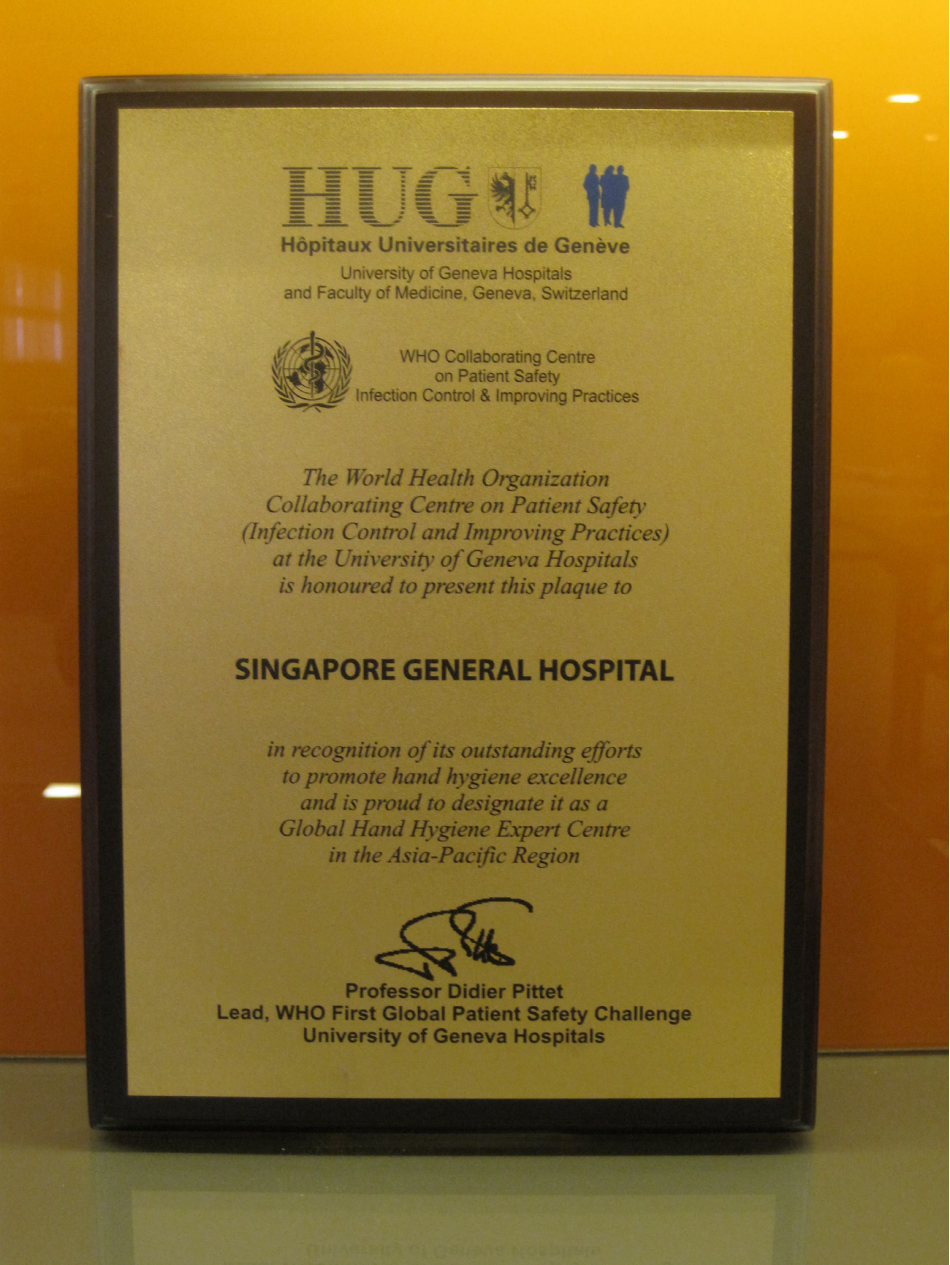
**Singapore General Hospital received status as Global Hand Hygiene Expert Centre**.

Leadership's support of the program evidenced through visible leadership presence, messaging and release of resources is the key factor in helping to make the program a true success. The hospital was recognised as a Global Hand Hygiene Expert Centre in January 2011 (Figure 14). The use of the WHO multi-prong interventions is successful in improving hand hygiene compliance with concomitant reduction in healthcare-associated infections.

## Competing interests

We have received support of our hand hygiene program from the following companies:

1. B. Braun

2. Schülke

3. Mölnlycke Health Care

## Authors' contributions

MLL was the advisor to the hand hygiene program. KBH was the coordinator in the hand hygiene program. All authors have read and approved this manuscript for publication.

## References

[B1] WHO Guidelines on Hand Hygiene in Healthcare, WHO 2009 19582963

[B2] WHO Guidelines on Hand Hygiene in Healthcare (Advanced Draft), WHO 2006

[B3] 5 Million Lives Campaign Getting Started Kit: Reduce Methicillin-Resistant Staphylococcus aureus (MRSA) Infection How-to Guide 2008 Cambridge, MA: Institute for Healthcare Improvement http://www.ihi.org

